# Stable DOPG/Glycyrrhizin Vesicles with a Wide Range of Mixing Ratios: Structure and Stability as Seen by Scattering Experiments and Cryo-TEM

**DOI:** 10.3390/molecules26164959

**Published:** 2021-08-16

**Authors:** Carina Dargel, Friederike Gräbitz-Bräuer, Ramsia Geisler, Pascal Fandrich, Yvonne Hannappel, Lionel Porcar, Thomas Hellweg

**Affiliations:** 1 Physical and Biophysical Chemistry, Bielefeld University, Universitätsstr. 25, 33615 Bielefeld, Germany; cdargel@uni-muenster.de (C.D.); friederike.graebitz@uni-bielefeld.de (F.G.-B.); geisler@fkp.tu-darmstadt.de (R.G.); p.fandrich@uni-bielefeld.de (P.F.); yvonne.hannappel@uni-bielefeld.de (Y.H.); 2 Institut Laue-Langevin, 71 Avenue des Martyrs CS 20156, CEDEX 9, 38042 Grenoble, France; porcar@ill.eu

**Keywords:** DOPG, glycyrrhizin, small unilamellar vesicle (SUV), SAXS and SANS, WAXS

## Abstract

Phosphatidylglycerols represent a large share of the lipids in the plasmamembrane of procaryotes. Therefore, this study investigates the role of charged lipids in the plasma membrane with respect to the interaction of the antiviral saponin glycyrrhizin with such membranes. Glycyrrhizin is a natural triterpenic-based surfactant found in licorice. Vesicles made of 1,2-dioleoyl-*sn*-glycero-3-phospho-rac-(1’-glycerol) (DOPG)/glycyrrhizin are characterized by small-angle scattering with neutrons and X-rays (SANS and SAXS). Small-angle scattering data are first evaluated by the model-independent modified Kratky–Porod method and afterwards fitted by a model describing the shape of small unilamellar vesicles (SUV) with an internal head-tail contrast. Complete miscibility of DOPG and glycyrrhizin was revealed even at a ratio of lipid:saponin of 1:1. Additional information about the chain-chain correlation distance of the lipid/saponin mixtures in the SUV structures is obtained from wide-angle X-ray scattering (WAXS).

## 1. Introduction

**Figure 1:**
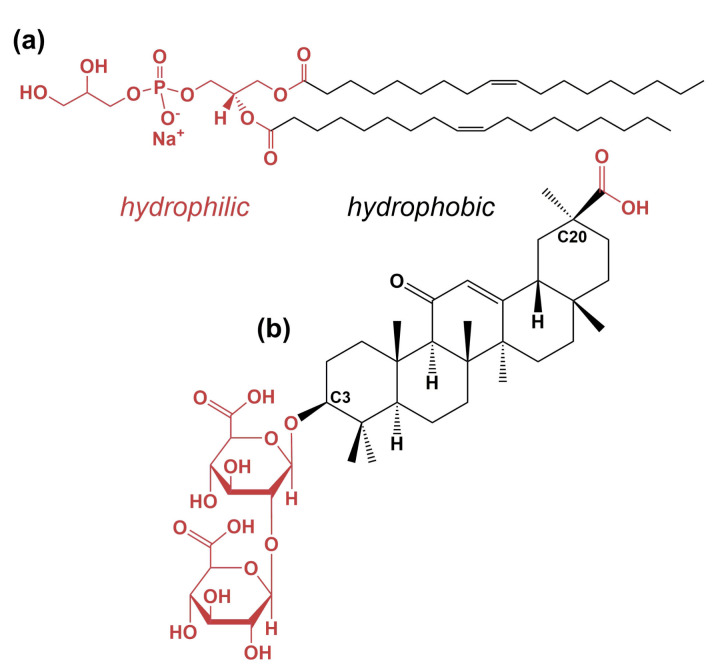
Molecular structures of (**a**) the phospholipid 1,2-dioleoyl-*sn*-glycero-3-phospho-rac-(1’-glycerol) (DOPG) and, (**b**) the saponin glycyrrhizin. Hydrophilic molecular parts are shown in red, hydrophobic ones in black. In this work, glycyrrhizin is present in the fully deprotonated state.

Saponins are amphiphilic molecules which are found in a large variety in plants, granting them the name bio-surfactants [1,2,3,4]. The hydrophobic backbone of saponins is a steroid or triterpene, to which a different number of hydrophilic sugar chains is attached [1,5]. The saponin used in this study is glycyrrhizin (see Figure 1b), which can be extracted from the roots of *Glycyrrhiza glabra*, also known as licorice [6]. Glycyrrhizin is commonly used as a sweetener because it is 30–50 times sweeter than glucose and exhibits a low toxicity [7,8,9]. Nevertheless, the recommended daily consumption is less than 0.229 mg glycyrrhizin/kg body weight/day [10]. Like many other saponins [1,2,11,12], glycyrrhizin exhibits several pharmacological actions, such as an anti-inflammatory, an antimicrobial and -viral, an antioxidative and an antitumor activities [9,13]. Particularly, glycyrrhizin inhibits the replication of the SARS-CoV associated coronavirus [14] and therefore might be a candidate for the treatment of COVID-19 [15,16]. In Japan, glycyrrhizin is already used for the treatment of different types of hepatitis [17,18]. In this context, vesicles carrying glycyrrhizin are also promising for the delivery of saponin [19]. Moreover, such systems might also allow the treatment of inflammations of the skin [20].

Due to its acidic groups, glycyrrhizin is more correctly called glycyrrhizinic acid [7]. The hydrophobic backbone of this molecule is based on a triterpene called glycyrrhetinic acid [9,21]. The sugar-based, hydrophilic part of the molecule is attached to the backbone via the C3 position and is built by two glucuronic acid molecules (see Figure 1b). An additional acidic group attached to the C20 position of glycyrrhetinic acid significantly influences the amphiphilicity of the whole molecule as a function of pH. The acidic groups are mostly protonated up to a pH value of 6 and a clear critical micelle concentration (cmc) can be found for pH < 6. At pH > 6, glycyrrhizin does not self-assemble into discrete aggregates anymore [21]. Here, deprotonation of the acidic groups causes a loss of the clear amphiphilic character and the negative charge induces repulsion effects between different glycyrrhizin molecules [22]. Moreover, deprotonation of the acidic groups leads to a strong increase in solubility in aqueous solution [21]. In water, glycyrrhizin shows an interesting self-assembly behavior, which was shown by AFM experiments [23].

Wojciechowski et al. proved weak interactions of glycyrrhizin with lipid membranes [24,25]. MD simulations for DPPC (1,2-dipalmitoyl-*sn*-glycero-3-phosphocholine) and DOPC (1,2-dioleoyl-*sn*-glycero-3-phosphocholine) based bilayers, conducted by Selyutina et al., suggested that glycyrrhizin is most likely completely incorporated into the lipid bilayer. In the case of DPPC, a thinning of the membrane is attributed to the incorporation of glycyrrhizin into the hydrophobic interior of the lipid bilayer [26]. According to Selyutina et al., pore formation induced by glycyrrhizin addition leads to a higher permeability of cell membranes, which leads to an enhanced effect of drugs [27,28]. MD simulations conducted by Shelepova et al. did not confirm such a pore formation process [29]. Hence, the exact mechanism causing the improved permeability remains unknown.

Previous work on the interaction of glycyrrhizin with model membranes composed of the phospholipid 1,2-dimyristoyl-*sn*-glycero-3-phosphocholine (DMPC) clearly revealed an interaction of the saponin and the lipid [30]. At saponin amounts higher than ≈40 mol%, the DMPC model membrane is completely decomposed into smaller, free-standing bilayer fragments in the form of bicelles. This decomposition is temperature-dependent and occurs at temperatures well below the lipids $T_{m}$; thus, when the lipid is present in its gel phase. Similar observations were made by Geisler et al. for the saponin β-aescin [31,32]. In the lipids’ liquid crystalline phase (at $T>T_{m}$), the formation of correlated membrane structures was shown. This indicates, that glycyrrhizin might not be entirely incorporated into the hydrophobic membrane part or that an interaction between the saponin molecules is nevertheless possible. However, these studies are limited to the phospholipid DMPC and analysis was performed depending on its phase behavior.

We want to extend the investigation of the interaction of glycyrrhizin with model membranes to another phospholipid class. For this purpose, we chose the negatively charged phospholipid 1,2-dioleoyl-*sn*-glycero-3-phospho-rac-(1’-glycerol) (DOPG), which belongs to the class of phosphatidylglycerols (PGs). PG lipids are widely distributed in the plasmamembrane of prokaryotes such as microorganisms [33,34]. In eukaryotic, mammalian systems, only minor amounts of PGs are found [33,35]. In these systems, PGs are mainly present and synthesized in the mitochondria as a precursor for cardiolipin, a lipid only located in the inner mitochondrial membrane and essential for function of many enzymes involved in the mitochondrial metabolism [36]. Moreover, in photosynthetic membranes of higher plants or algae and cyanobacteria, PG introduces a negative charge essential for proper assembly of the photosynthesis apparatus [37,38,39].

DOPG (see Figure 1a) is used for the formation of a negatively charged double-layered model membrane in the form of small unilamellar vesicles (SUVs). The studies of Claessens et al. and Esseling-Ozdoba et al. showed that formation of long term stable DOPG SUVs is possible from this lipid [40,41]. Under conventional experimental conditions, DOPG always adopts the liquid crystalline phase due to its low $T_{m}$ of −18 ${}^{\circ }$C [42,43,44]. In general, membranes composed of PG lipids are much less investigated than lipid membranes based on phosphatidylcholines (PCs). A tendency of formation of asymmetric bilayers by PGs was found [45,46] and PG membranes are thicker than the corresponding PC membranes [47]. In PG membranes, hydrogen bonding between the glycerol and phosphate moieties results in a shielding of the negative charges at the bilayer surface [48].

This study elaborates the influence of added glycyrrhizin to long term stable DOPG vesicles. For this purpose, mixtures of DOPG with glycyrrhizin up to a ratio of 1:1 are extruded to generate SUVs. These high amounts of glycyrrhizin are used, because for a system composed of DMPC and glycyrrhizin a complete membrane solubilization was observed at ratios of ≈1:1. First, the general shapes of the structures formed are analyzed by cryogenic transmission electron microscopy (cryo-TEM) in the case of pure DOPG and a sample with the highest glycyrrhizin content of 50 mol%. Afterwards, several scattering methods are employed to characterize the SUV size parameters. Wide-angle X-ray scattering (WAXS) resolves the glycyrrhizin content dependent acyl-chain correlation distance. Small-angle scattering with neutrons and X-rays (SANS/SAXS) is used to determine the overall size, membrane thickness, and membrane contrast profile. The evaluation of the data is performed by model-independent as well as model-dependent analyses. The overarching aim of this study is to gain a deeper understanding of the interaction of glycyrrhizin with model membranes mimicking procaryotic cells.

## 2. Materials and Methods

### 2.1. Chemicals and Sample Preparation

The phospholipid DOPG was obtained from Lipoid GmbH (purity: ≥99%, Ludwigshafen, Germany). The saponin glycyrrhizin (used as ammonium salt, ≥95%, CAS: 53956-04-0), chloroform and deuteriumoxide (D_2_O) were purchased from Sigma-Aldrich (Munich, Germany). Aqueous samples were prepared with purified water (Sartorius arium VF pro, Göttingen, Germany). For all samples a 50 mM phosphate buffer with a pH/pD value of 7.4 in D_2_O/H_2_O was used [49].

The lipid mass concentration was fixed for all samples to a value of 15 g·L${}^{-1}$. The glycyrrhizin contents $x_{\mathrm{glycyrrhizin}}$ range from 0 to 50 mol%, with respect to the lipid concentration:

(1) $$x_{\mathrm{glycyrrhizin}}=\frac{n_{\mathrm{glycyrrhizin}}}{n_{\mathrm{DOPG}}+n_{\mathrm{glycyrrhizin}}}.$$

For the preparation of DOPG-glycyrrhizin mixtures DOPG was dissolved in chloroform and dried using a rotary evaporator. To remove residuals of chloroform from the thin lipid film, the sample was stored over night at 60 ${}^{\circ }$C. After that, the lipid film was rehydrated with the glycyrrhizin-containing buffer solution at the desired glycyrrhizin concentration. Due to its acidic functions and the decrease in pH, small amounts of concentrated sodium hydroxide were added to adjust the pH value of the glycyrrhizin stock solution to 7.4. After rehydrating the lipid film, the samples were subjected to five consecutive freeze–thaw cycles (in liquid nitrogen and warm water) and extruded (with increasing $x_{\mathrm{glycyrrhizin}}$) through a membrane with a pore size of 500 Å (Whatman, Avanti Polar Lipids Inc., Alabaster, AL, USA) using a conventional extruder (at least 21 passes, extruder from Avanti Polar Lipids Inc., Alabaster, USA). For each solvent, a new membrane was used.

### 2.2. Cryogenic Transmission Electron Microscopy (Cryo-Tem)

By cryo-TEM, the structures formed by pure DOPG and DOPG with the highest glycyrrhizin amount of 50 mol% prepared in D_2_O-based buffer were visualized. For imaging, a JEOL JEM-2200FS electron microscope (JEOL, Freising, Germany) equipped with a cold field emission electron gun was used. The sample was applied to a lacey carbon film coated grid (200 Mesh, Cu, Science Services GmbH, Munich, Germany) and vitrified by a Leica blotting and plunging device operated at room temperature (≈25 ${}^{\circ }$C) (Leica EM GP, Leica Mikrosysteme Vertrieb GmbH, Wetzlar, Germany). The samples were plunged into liquid ethane, which was cooled with liquid nitrogen to achieve sufficiently fast cooling and freezing without formation of ice crystals. After freezing, the grids were transferred to a cryo transfer and tomography holder (Fischione, Model 2550, E.A. Fischione Instruments, Pittsburgh, PA, USA). The microscope was operated at an acceleration voltage of 200 kV and the images were recorded digitally by a bottom mounted camera (Gatan OneView, Gatan, Pleasanton, CA, USA). The images were afterwards processed with a digital imaging processing program (Digital Micrograph^®^, Version 3.21, GMS 3, Gatan, Pleasanton, CA, USA).

### 2.3. Small-Angle Scattering (SAS)

Small-angle scattering (SAS) resolves the structure of colloidal particles (for example, dispersed in solution) and of, e.g., vesicular systems [50,51,52,53].

The scattered radiation is recorded at an angle of 2θ and is commonly converted into the magnitude of the scattering vector *q*. This allows one to conduct different scattering experiments—for instance, using different kinds of radiation (characterized by the wavelength λ), to a common scale (see Equation (2)).

(2) $$\;q=\frac{4\pi n}{\lambda }\cdot \sin\left(\theta \right)$$

The total scattering intensity is given by

(3) $$\;I\left(q\right)=N\cdot {\left(\Delta \rho \right)}^{2}\cdot V^{2}\cdot P\left(q\right)\cdot S\left(q\right).$$

This depends on the number of particles *N*, the particles scattering volume *V*, the form factor P(q) and the structure factor S(q). S(q) is assumed to be negligible due to the low mass concentration of lipid and saponin used in solution. The scattering length density difference Δρ, or ΔSLD, strongly depends on the radiation used. Whereas X-rays highlight the electron dense membrane parts (the head groups), neutrons highlight the protonated parts of the lipid membrane. Thus, by neutrons the head and tail part of the membrane cannot be separated that clearly and the complementary usage of both kinds of radiation provides more detailed information on the system.

In this study, model-independent and model-based approaches are applied to gain structural information on the system investigated. The model-independent modified Kratky–Porod (MKP) [54] method is used to determine the membrane thickness from SANS and SAXS data [55]. In this method, $I\left(q\right)q^{4}$ is plotted as function of *q* and the resulting signal is approximated with a 4th order polynomial function to determine the maximum position $q_{\max,\mathrm{MKP}}$ [54,56]. From the position $q_{\max,\mathrm{MKP}}$ the membrane thickness $d_{m,\mathrm{MKP}}$ can be calculated using the relation $q_{\max,\mathrm{MKP}}$·$d_{m,\mathrm{MKP},}\;\mathrm{SAXS}=2\pi$ in the case of SAXS [55] and $q_{\max,\mathrm{MKP}}$·$d_{m,\mathrm{MKP},}\;\mathrm{SANS}=\pi$ in the case of SANS [57].

Additionally, structural parameters of the SUVs are derived from model-based fitting with a core multishell sphere (CMS) describing a hollow sphere with head-to-tail contrast in the sphere-building lipid membrane [58]. The CMS model is implemented in the program SASView [59] and the SUV size expressed as the core radius $R_{c}$, its polydispersity $\sigma_{R_{c}}$, the membrane thickness $d_{m}$, and the corresponding polydispersity $\sigma_{d_{m}}$ are determined. A more precise description of the model can be found in Section 3.3.2.

#### 2.3.1. Small-Angle Neutron Scattering (SANS)

SANS experiments were performed with samples prepared in D_2_O buffer. Measurements were performed using the D22 instrument at the Institut Laue-Langevin (ILL) in Grenoble (France). The samples were filled into 2 mm quartz cuvettes (Hellma Analytics, Müllheim, Germany) and measured in a 15-position sample holder at a temperature of 30 ${}^{\circ }$C. A *q*-range from 1.7 × 10${}^{-3}$ Å${}^{-1}$ to 0.4 Å${}^{-1}$ was covered using a neutron wavelength of 6 Å for the sample to detect distances of 2.8 m and 17.6 m, and a neutron wavelength of 12 Å at 17.6 m. The wavelength resolution was Δλ/λ=10 %. Initial treatment of the 2D data was carried out with the software GRASP provided by the ILL [60]. The data were reduced with respect to empty cell, background, transmission and direct beam measurement to finally obtain the circularly averaged absolute intensity.

#### 2.3.2. Small-Angle X-ray Scattering (SAXS)

DOPG samples prepared in D_2_O as well as in H_2_O buffer were measured on an inhouse SAXS/WAXS system (XEUSS, Xenocs, Sassenage, France) equipped with a CuK_α_ source (λ = 1.541 Å, GeniX Ultra low divergence, Xenocs) and a Pilatus 300K hybrid pixel detector (Dectris, Baden Deattwil, Switzerland). The samples were measured to detect distances of 2.7 m and 0.8 m convering a *q*-range from 6·10${}^{-3}$ Å${}^{-1}$ to 0.4 Å${}^{-1}$. The 2D data were analyzed using the Foxtrot software (V3.3.4) [61]. The samples were measured in a flow-through Kapton capillary (1 mm, GoodFellow GmbH, Bad Nauheim, Germany) positioned in a Linkam stage (Linkam Scientific, Tadworth, UK) at a temperature of 30 ${}^{\circ }$C. The scattering of the sample was normalized with respect to incident intensity, sample thickness, acquisition time, transmission and background. The data were brought to absolute scale using glassy carbon type 2 as standard [62]. After normalization, the data were treated by the dynamic rebin formalism implemented in the program SAXSutilities to improve statistics at high *q*-values (min. steps: 1, min. Δq: 0.005 Å^−1^) [63].

### 2.4. Wide-Angle X-ray Scattering (WAXS)

Wide-angle X-ray scattering was used to determine the chain–chain correlation distance $d_{\mathrm{WAXS}}$ in the lipid bilayers. The correlation signal observed in WAXS occuring at the position $q_{\mathrm{WAXS}}$ is directly related to $d_{\mathrm{WAXS}}$ via $d_{\mathrm{WAXS}}=2\pi /q_{\mathrm{WAXS}}$ [64]. WAXS measurements for samples prepared in D_2_O as well as in H_2_O buffer were also performed on the XEUSS SAXS/WAXS setup (see Section 2.3.2 for experimental details and data reduction). All measurements were performed at a temperature of 30 ${}^{\circ }$C at a sample to detect distance of 0.16 m, covering a *q*-range from 0.5 Å${}^{-1}$ to 2 Å${}^{-1}$.

## 3. Results

### 3.1. General Phase Behavior and Identification of Particle Shape by Cryo-TEM

**Figure 2:**
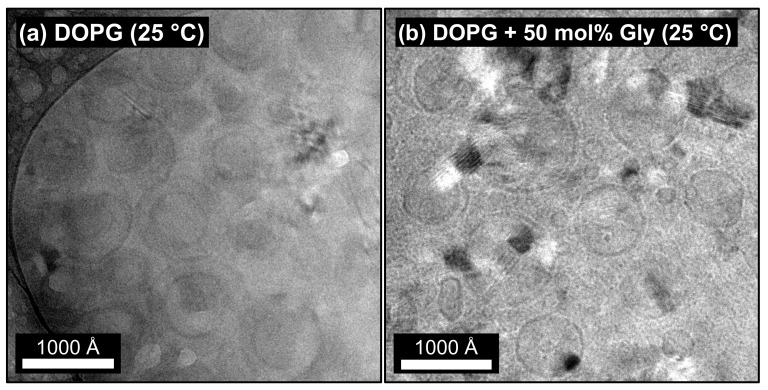
Cryo-TEM images of (**a**) DOPG and (**b**) DOPG with 50 mol% glycyrrhizin in D_2_O as solvent. The samples were kept at room temperature (≈25 ${}^{\circ }$C) before freezing. In both cases, the typical pattern of unilamellar vesicles is observed.

As described in Section 2.1, DOPG SUVs were prepared in presence of glycyrrhizin using D_2_O and H_2_O buffer, respectively. Photographs of the sample vials are shown in Figure S1 in the Electronic Supplementary Materials. Independent of $x_{\mathrm{glycyrrhizin}}$, all samples, in D_2_O as well as in H_2_O buffer, exhibit a bluish color and do not show any precipitation even after a period of at least six months, even at the highest glycyrrhizin content. Hence, long term stable SUVs are formed. To verify the SUV structure, cryo-TEM imaging was performed on a sample composed of pure DOPG and a sample with $x_{\mathrm{glycyrrhizin}}$ = 50 mol%, both prepared in D_2_O buffer. Figure 2a shows the image for pure DOPG and (b) for a 1:1 mixture of DOPG and glycyrrhizin.

In both cases, a circular particle cross-section is visible, which clearly indicates the formation of SUVs. For pure DOPG, in most cases a smaller vesicle is surrounded by a bigger one. Although cryo-TEM images of DOPG recorded by Esseling-Ozdoba [41] show a similar phenomenon, we believe that this feature might be an artifact of the usage of D_2_O as solvent and/or the freezing process. The average SUV diameter for pure DOPG vesicles is ≈500–900 Å. In comparison with pure DOPG SUVs, the sample with 50 mol% glycyrrhizin does not exhibit ’nested’ vesicles. Moreover, in addition to vesicles with a similar diameter of ≈600–800 Å (compared to pure DOPG), very small vesicles can also be seen. Appearance of these vesicles might be attributed to the freezing process. Existence of such small vesicles was not directly proven by small-angle scattering, but a high polydispersity is found for high amounts of glycyrrhizin by this method (see Section 3.3.2). In addition to the samples presented in Figure 2, pure DOPG and a sample containing 50 mol% glycyrrhizin prepared in H_2_O buffer and diluted to a DOPG mass concentration of 0.5 g·L${}^{-1}$ were recorded (see Figure S2a,b). In both cases the SUV structure was preserved even after dilution. For the glycyrrhizin-containing sample, we want to mention that the effective glycyrrhizin content might be changed. The saponin has a much higher monomeric solubility in aqueous solution compared to the lipid [65]. Hence, glycyrrhizin might have been partially removed from the DOPG membrane by dilution. A determination of the effective concentration of the glycyrrhizin in the DOPG membrane would greatly advance the interpretation of the behavior of the present system. However, the system behaves highly dynamic in terms of changes such as dilution or changes in temperature. For this reason it has unfortunately not been possible to date to determine the effective saponin concentration in the DOPG membrane under variable conditions.

### 3.2. Influence of Glycyrrhizin on the Chain–Chain Correlation Distance $d_{\mathrm{WAXS}}$ in DOPG Membranes Resolved by WAXS

**Figure 3:**
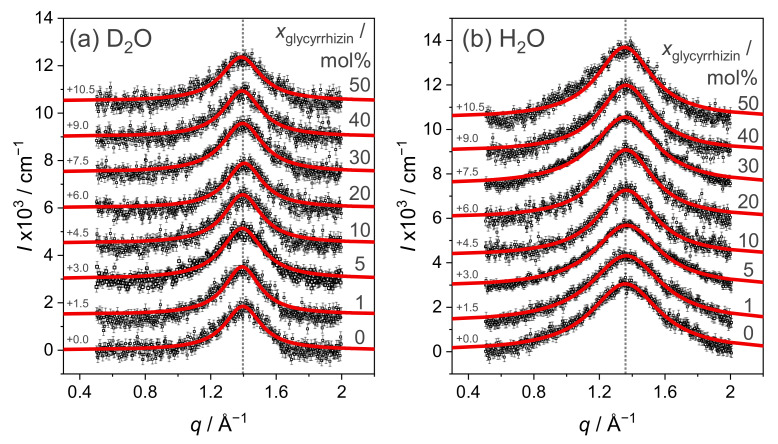
WAXS signal of mixtures of DOPG and glycyrrhizin in (**a**) D_2_O and (**b**) H_2_O buffer. The glycyrrhizin content is represented by the numbers on the right. For better readability, the data are baseline shifted by the gray values on the left. Red lines are Lorentzian fits. Dotted lines indicate the position of $q_{\mathrm{WAXS}}$ in the absence of glycyrrhizin.

Information on the $x_{\mathrm{glycyrrhizin}}$-dependent modifications of the lipids chain–chain correlation distance can be obtained from WAXS measurements (some authors call this parameter the headgroup distance). WAXS signals obtained for SUVs composed of DOPG and glycyrrhizin up to a content of 50 mol% are shown in Figure 3a for the D_2_O and (b) for the H_2_O based buffer. The corresponding real space distances $d_{\mathrm{WAXS}}$ for both solvents are listed in Table 1. It is expected that incorporated glycyrrhizin will, at least at higher contents, significantly contribute to the WAXS signal. Similar observations were made for the incorporation of aescin into DMPC bilayers [66,67].

The broad shape of the signals in Figure 3 indicates, as expected, that DOPG adopts the fluid crystalline phase [66]. Moreover, shape and position of the signals for pure DOPG in D_2_O and H_2_O buffer resemble the WAXS signal reported by Caracciolo et al., for pure DOPG [68]. Compared to that study, a similar chain–chain correlation distance of ≈4.5 Å for D_2_O buffer and ≈4.6 Å for H_2_O buffer is obtained for pure DOPG. The small difference in $d_{\mathrm{WAXS}}$ for both solvents might be induced by an altered hydration of the lipids by D_2_O and H_2_O or an altered viscosity due to the usage of the different solvents. Addition of glycyrrhizin to DOPG does not induce a change in the shape of the WAXS signal even at high glycyrrhizin contents. Consequently, $d_{\mathrm{WAXS}}$ also does not show a significant evolution with varying $x_{\mathrm{glycyrrhizin}}$ and remains around 4.5–4.6 Å for D_2_O and H_2_O buffer.

Several simulation studies in literature suggest that glycyrrhizin is fully incorporated into the hydrophobic part of the membrane in case of the lipids DPPC or DOPC [26,29,69]. Additionally, at a ratio of lipid:saponin of 1:1 a modification of the WAXS signal in comparison to pure DOPG is not observable. This can probably be explained by similar dimensions (see Table S2) and therefore also similar molecule-molecule packing distances of DOPGs hydrophobic part and glycyrrhizin in the SUV structures. Even formation of glycyrrhizin clusters would therefore not necessarily lead to a modification of the WAXS signal in the present case.

### 3.3. Characterization of SUV Structure by SAS

**Figure 4:**
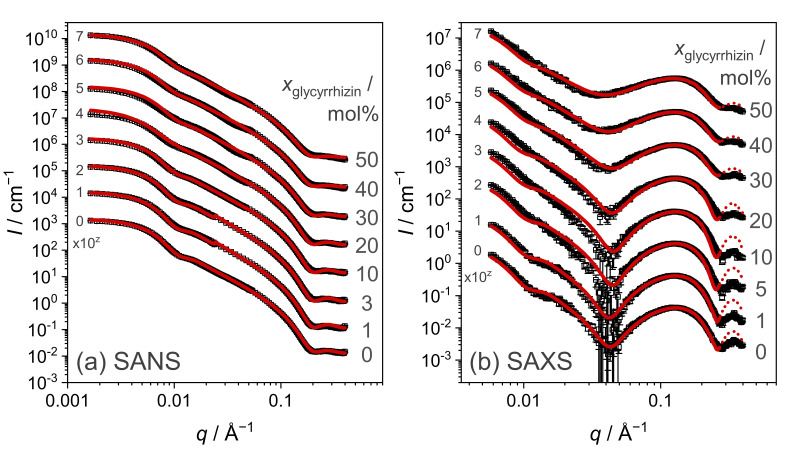
Comparison of (**a**) SANS and (**b**) SAXS data for samples with different $x_{\mathrm{glycyrrhizin}}$ in D_2_O buffer. $x_{\mathrm{glycyrrhizin}}$ is denoted by numbers on the right. Solid lines are approximations with the CMS model from SASView [59]. For better readability, the data are scaled by different multiples of 10 (exponent *z*) indicated by the numbers on the left side of the curves. Dotted lines in panel (**b**) are extrapolations of the CMS fits beyond the fit range.

Modifications of the overall SUV structures and especially the lipid membrane upon glycyrrhizin addition are studied by SANS and SAXS. Scattering curves for samples with different $x_{\mathrm{glycyrrhizin}}$ prepared in D_2_O buffer are shown in Figure 4a for neutrons and Figure 4b for X-rays, respectively. SAXS curves for samples in H_2_O buffer are additionally shown in Figure S3 in comparison with the data recorded for samples in D_2_O buffer.

The shapes of the SANS curves (Figure 4a) are similar for all $x_{\mathrm{glycyrrhizin}}$ and clearly indicates presence of SUV structures [52,55]. A tendency to form correlated membrane structures is not discernible, which indicates that a contact of glycyrrhizin between different vesicles does not occur. The SAXS curves for both solvents show a prominent membrane contribution around 0.1 Å^−1^ (see Figure 4b and Figure S3). With increasing $x_{\mathrm{glycyrrhizin}}$, the minimum of the scattering curves at q≈ 0.04 Å^−1^ smears, which indicates a change of the membrane contrast and/or its polydispersity seen by X-rays induced by glycyrrhizin incorporation. Glycyrrhizin-induced changes of the membrane thickness will first be evaluated by the model-independent MKP method and afterwards verified by model-dependent fitting of the SUV structures.

#### 3.3.1. Model-Independent Evaluation of SAS Data

**Figure 5:**
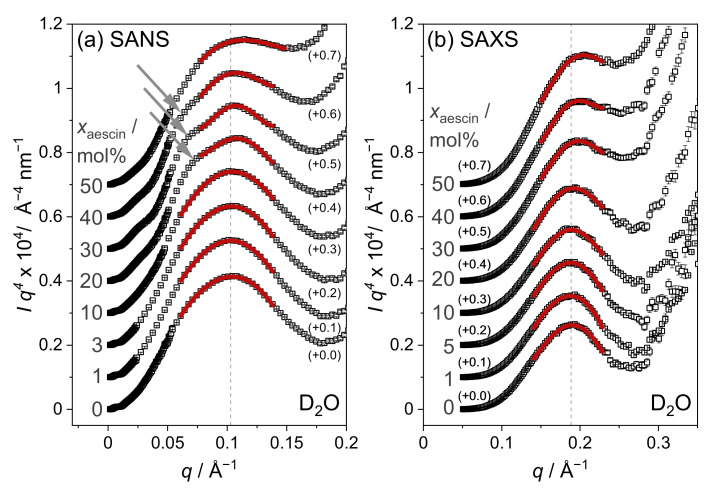
Modified Kratky–Porod plots of (**a**) SANS and (**b**) SAXS data obtained for samples with different $x_{\mathrm{glycyrrhizin}}$ in D_2_O as solvent. Solid lines are 4th order polynomial approximations. The maximum of these polynomial fits was used to determine the membrane thickness $d_{\mathrm{MKP}}$. A shoulder in the signal seen by SANS for samples with $x_{\mathrm{glycyrrhizin}}$ between 20 mol% and 40 mol% is highlighted by gray arrows. The glycyrrhizin content $x_{\mathrm{glycyrrhizin}}$ is denoted by numbers on the left. For better readability of the figure, the data are baseline-shifted by the numbers in brackets.

**Figure 6:**
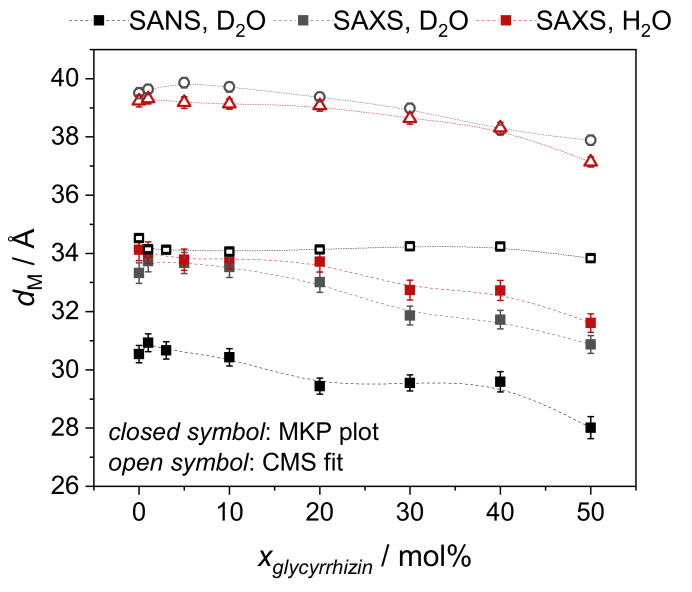
Membrane thicknesses obtained from model-dependent CMS fitting as well as from model-independent MKP approximation for samples in D_2_O buffer measured by SANS ($d_{D_{2}O,\mathrm{SANS}}$) and SAXS ($d_{D_{2}O,\mathrm{SAXS}}$) and samples in H_2_O buffer measured by SAXS ($d_{H_{2}O,\mathrm{SAXS}}$).

The MKP method [54,56] is used to determine the membrane thickness of the mixed DOPG-glycyrrhizin SUVs. A plot of $I\left(q\right)q^{4}$ as a function of *q* is shown in Figure 5 for samples in D_2_O buffer investigated by SANS and SAXS. Equivalent data for samples prepared in H_2_O buffer are shown in Figure S4. Membrane thicknesses $d_{m,\mathrm{MKP}}$ for all samples investigated by SANS and SAXS obtained from the position of maximum intensity are shown as function of $x_{\mathrm{glycyrrhizin}}$ in Figure 6 and are additionally listed for comparison in Table S1. The MKP plots for data recorded by SANS show a shoulder at *q*≈0.6 Å^−1^ especially for $x_{\mathrm{glycyrrhizin}}$ between 20 mol% and 40 mol% (see gray arrow in Figure 5). This shape deformation indicates a change in the membrane composition in the case of the homogeneous SANS contrast, which is not directly visible from the bare scattering data (see Figure 4).

As expected, the membrane thicknesses obtained by SAXS for samples in D_2_O and H_2_O buffer do not differ significantly (especially for low $x_{\mathrm{glycyrrhizin}}$) and $d_{m,\mathrm{MKP}}$ decreases slightly with increasing $x_{\mathrm{glycyrrhizin}}$. Values determined by SAXS are ≈3–4 Å higher compared to the ones obtained from SANS. This seems reasonable because different membrane contrasts do not lead to exactly the same membrane thickness values. Pencer et al. [57] also determined the membrane thickness of DOPG by the MKP method from SANS data and reported a value of 31.29 ± 0.05 Å. This value is similar to the one obtained in our study with a value of 30.5 ± 0.3 Å. Moreover, in the study of Pencer et al., it is stated that the thickness obtained from the MKP method corresponds to the thickness of the hydrocarbon region [57] and therefore appears smaller than the total membrane thickness.

#### 3.3.2. Model-Dependent Fitting of SAS Data

**Figure 7:**
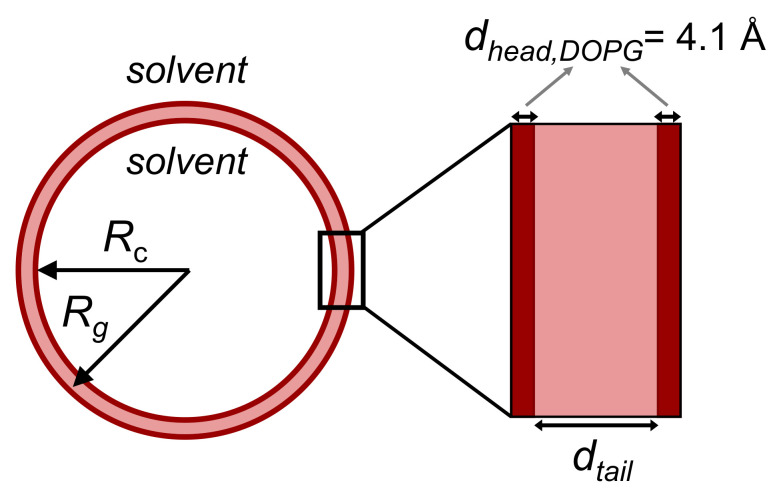
Schematic representation of a SUV structure and parameters obtainable from the CMS model. Different locations of radii obtained from different methods ($R_{c}$ and $R_{g}$) are depicted. Moreover, size parameters describing the lipid membrane are shown.

In this section, the scattering curves obtained from SANS and SAXS are evaluated by model-dependent fitting to derive the SUV size, membrane thickness and membrane contrast parameters. To describe a SUV shape with a head-to-tail contrast over the lipid membrane, the core multishell (CMS) model with three shells implemented in the program SASView [59] was used. This model was already successfully used to model the SUV shape in mixtures of the phospholipid DMPC and the saponin aescin [58,70,71]. Parameters obtainable from this model are the core radius of the SUV $R_{c}$, the corresponding polydispersity $\sigma_{R_{c}}$ and the thicknesses of the lipids head $d_{\mathrm{head}}$ and tail part $d_{\mathrm{tail}}$. The latter parameter can also be accompanied by a polydispersity $\sigma_{d_{\mathrm{tail}}}$. The total membrane thickness amounts to $d_{M,\mathrm{CMS}}=2$·$d_{\mathrm{head}}+d_{\mathrm{tail}}$. A scheme describing the model used is shown in Figure 7. Moreover, parameters expressing the membrane contrast can be obtained from the model. Thereby, $SLD_{\mathrm{head}}$ describes the scattering length density of the lipids head part and $SLD_{\mathrm{tail}}$ the one of the membrane interior. Moreover, $SLD_{\mathrm{solvent}}$, found in the interior as well as exterior of the SUV, relates the scattering length density of the solvent with the one of the lipid membrane. Some of the mentioned parameters were calculated prior to fitting and other parameters were optimized by the fitting procedure.

From comparisons to the literature, it was assumed that the glycyrrhizin molecules are completely incorporated into the hydrophobic part of the membrane [26,29,69] and that the membranes hydrophilic part therefore only consists of DOPG head groups. The size of the DOPG head group $d_{\mathrm{head}}$ was fixed to a value of 4.1 Å based on a study of Pan et al. [53]. This value is also similar to the size of a glycerol-based lipid head group determined by Kučerka et al. [72] with a value of 4.3–4.9 Å. Additionally, $SLD_{\mathrm{head}}$ and $SLD_{\mathrm{solvent}}$ ($NSLD_{D_{2}O}$ = 6.36 × 10^−6^ Å^−2^, $XSLD_{D_{2}O}$ = 9.4 × 10^−6^ Å^−2^ and $XSLD_{H_{2}O}$ = 9.43 × 10^−6^ Å^−2^) were calculated and fixed prior to fitting. For $SLD_{\mathrm{head}}$, again the study of Pan et al. [53] was used to determine $SLD_{\mathrm{head}}$ of DOPG for a temperature of 30 ${}^{\circ }$C. In this study, the volumes of the whole DOPG molecule as well as the head group are reported. Based on this value and in anology to Sreij et al. [58], $SLD_{\mathrm{head}}$ was calculated for the usage of neutrons as well as X-rays (see Table S2). In the same table, the molecular volume and the resulting scattering length densities for the saponin glycyrrhizin are listed in comparison to the values for DOPG. In this case, the molecular volume was derived from the program ChemSketch [73].

**Figure 8:**
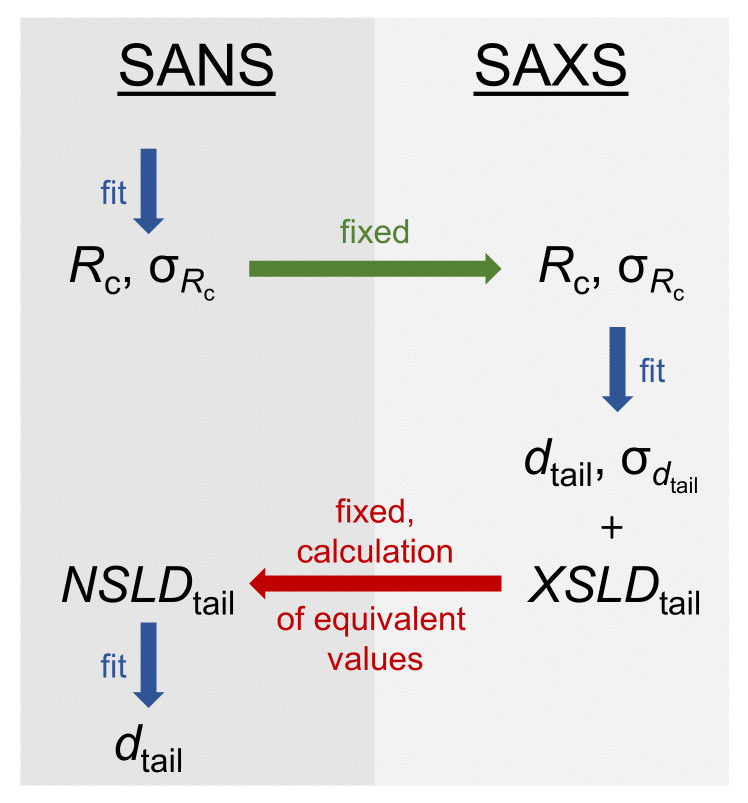
Flow chart showing the fitting procedure of equivalent SANS and SAXS data with the CMS model.

SAS scattering curves are shown in Figure 4 and solid lines depict the CMS fits. The procedure for determining the CMS fits is shown in Figure 8 in a flow chart. The overall size of the underlying structures seen in SANS and SAXS should be identical, because exactly the same samples were investigated. However, because the membrane thicknesses obtained from both methods are most likely different due to different contrasts (see membrane thickness from MKP evaluation in Figure 6), we determined independent parameter sets for SAXS and SANS. Due to a limited *q*-range in the case of SAXS, the SUV size and its polydispersity, expressed as $R_{c}$ and $\sigma_{R_{c}}$, were derived from the SANS data. To obtain correct values for $\sigma_{R_{c}}$, the wavelength resolution was taken into account in the fitting process. Both values ($R_{c}$ and $\sigma_{R_{c}}$) were afterwards fixed for the approximation of the SAXS data. Values obtained for $R_{c}$ and $\sigma_{R_{c}}$ are listed in Table 2. Thereby, a decrease in $R_{c}$ from 207 Å to 156 Å with increasing $x_{\mathrm{glycyrrhizin}}$ was observed, while $\sigma_{R_{c}}$ increases from 37 to 64 %. Here, we want to mention that the high value of $\sigma_{R_{c}}$ can have a significant influence on $R_{c}$. Especially for samples with $x_{\mathrm{glycyrrhizin}}$ of 0 and 1 mol%, the CMS fits follow the SAXS data nicely at low *q*, which indicates that determination of $R_{c}$ and $\sigma_{R_{c}}$ from SANS data yields reliable results (compare Figure 4).

In addition to the scattering data recorded in D_2_O buffer, SAXS measurements were performed on samples prepared in H_2_O buffer. The corresponding SAXS curves together with CMS fits are shown in Figure S3 in comparison to data obtained in D_2_O as solvent. CMS fitting of data from samples in H_2_O buffer was performed independently from samples prepared in D_2_O buffer, because different membranes are used for extrusion and additionally the solvent may have an influence on the SUV parameters. In Table S3, $R_{c}$ together with the $\sigma_{R_{c}}$-values were compared for SAXS data recorded in H_2_O buffer and SANS data recorded for samples prepared in D_2_O buffer. For pure DOPG SUVs, similar values for $R_{c}$ and $\sigma_{R_{c}}$ are obtained ($R_{c,\mathrm{SANS},D_{2}O}$ = 207 ± 2 Å, $\sigma_{R_{c,}\mathrm{SANS},D_{2}O}$ = 37 % and $R_{c,\mathrm{SAXS},H_{2}O}$ = 204 ± 7 Å, $\sigma_{R_{c,}\mathrm{SAXS},H_{2}O}$ = 32 %). In this case, a minimum in the SAXS curves quite clearly defines both parameters although a scattering plateau is not reached. With increasing $x_{\mathrm{glycyrrhizin}}$ and also increasing polydispersity $\sigma_{R_{c}}$ the size decrease for samples in H_2_O buffer is more pronounced, which might be due to the usage of the H_2_O.

The membrane contrast is sharper in the SAXS data and therefore the membrane SLD parameters were derived from this data after $R_{c}$ and $\sigma_{R_{c}}$ were fixed based on the results from SANS. For fitting the membrane part in the SANS data, equivalent NSLD values were calculated from the results for the XSLD values. Thereby, the molecular formula of a mixture of the DOPG tail part and the respective glycyrrhizin amount was taken into account. All NSLD and XSLD values for the hydrophilic and hydrophobic membrane parts are shown in Table S4.

As SLD and membrane size parameters are directly correlated, the thickness of the hydrophobic membrane part $d_{\mathrm{tail}}$ and therewith the complete membrane thickness $d_{M,\mathrm{CMS}}$ is determined during the SLD optimization process. Values for $d_{\mathrm{tail}}$ obtained from SANS and SAXS data for samples prepared in D_2_O/H_2_O buffer are listed in Table S5 and the corresponding values for $d_{M,\mathrm{CMS}}$ are plotted in Figure 6. For SANS, this parameter yielded only reasonable results after having fixed the NSLD values on the basis of the SAXS results. In the case of SAXS, a polydispersity of $d_{\mathrm{tail}}$ ($\sigma_{d_{\mathrm{tail}}}$) has to be considered to successfully represent the scattering data and especially the vanishing minimum at q≈ 0.04 Å^−1^. These values are additionally listed in Table S5.

As expected also from comparison with the MKP results, the thickness values derived from SAXS data for samples in D_2_O and H_2_O buffer are similar. In comparison to SAXS, the membrane thickness derived from SANS is about 4–5 Å lower. Pan et al. [53] determined a size of 27.5 Å for the hydrophobic part of the DOPG membrane. This value was derived from simultaneous fitting of SANS and SAXS data and lies between the values determined by us with both methods for pure DOPG vesicles ($d_{\mathrm{tail},\mathrm{SANS},D_{2}O\mathrm{DOPG}}$= 26.3 ± 0.1 Å and $d_{\mathrm{tail},\mathrm{SAXS},D_{2}O\mathrm{DOPG}}$ = 31.3 ± 0.1 Å). Simultaneous determination of $d_{\mathrm{tail}}$ was not performed in this work, because the results for SANS and SAXS from model-independent fitting showed a significant offset and having ’fixed’ $d_{\mathrm{tail}}$ from the more sensitive method SAXS leads to non-reliable results for the correlated NSLD values. The reason for this offset most probably is the different contrast seen by the different kinds of radiation used. At present SASView does not allow to account for this. However, we believe that simultaneous fitting of SAXS and SANS data will not lead to better results which would justify additional programming effort.

From Figure 6 as well as Table S5, a slight decrease in $d_{M,\mathrm{CMS}}$ with increasing $x_{\mathrm{glycyrrhizin}}$ can be concluded. This is in concordance with the MKP results. With a reduction in the membrane thickness of about 1–2 Å this change is rather small. A comparison with simulation studies of Selyutina et al. [26,69] showed that the present system behaves more similar to the DOPC-glycyrrhizin rather than the DPPC-glycyrrhizin system. The authors predicted that incorporation of glycyrrhizin into a DOPC membrane is not accompanied by a significant membrane thinning. As both membrane models contain double bonds in the hydrophobic membrane part and adopt the liquid crystalline phase, a similar observation for the present system seems reasonable.

**Figure 9:**
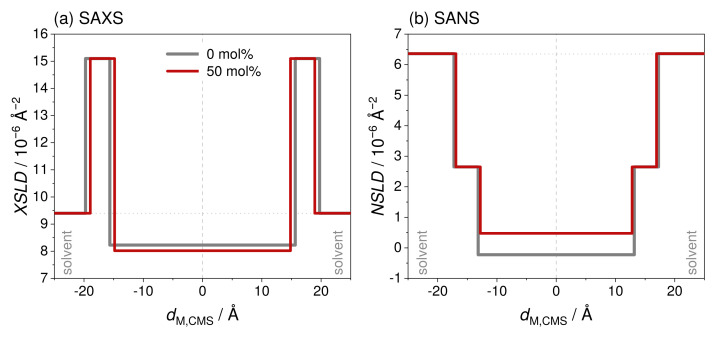
Scattering length density (SLD) profiles obtained from (**a**) SAXS and (**b**) SANS for DOPG SUVs without and with 50 mol% glycyrrhizin. The XSLD profiles were derived from fitting the SAXS data of samples prepared in D_2_O with the CMS model. NSLD values for the hydrophobic membrane part were calculated from the respective XSLD values taking the molecular formula of a mixture of the DOPG tail part and the respective glycyrrhizin amount into account.

Nevertheless, the weak membrane thinning effect is also visible in the SLD profiles which are compared in Figure 9 for both kinds of radiation and for samples prepared in D_2_O containing 0 and 50 mol% glycyrrhizin. In this figure, the XSLD profiles are shown in panel (a), whereas the NSLD profiles are presented in panel (b). A glycyrrhizin-induced change in the XSLD profile becomes visible, which can consequently also be seen in the NSLD profile. A decrease in the XSLD of the membranes hydrophobic part directly indicates incorporation of glycyrrhizin into the lipid membrane. With increasing $x_{\mathrm{glycyrrhizin}}$, the contrast between the membranes head and tail part increases (compare Table S4) and this observation does not directly explain the vanishing minimum at q≈ 0.04 Å^−1^. Therefore, the increased polydispersity of the hydrophobic membrane part might be the main factor for the vanishing intensity minimum in the SAXS curves (Figure 4 and Figure S3). For both solvents, $\sigma_{d_{\mathrm{tail}}}$ increases from ≈0–3 % to ≈9–13 % with increasing $x_{\mathrm{glycyrrhizin}}$ and upon glycyrrhizin incorporation (see Table S5). Due to the weaker head–tail contrast, it was not necessary to fit the polydispersity $\sigma_{d_{\mathrm{tail}}}$ to the SANS data and therefore a complementary value was not computed, since the number of parameters should be kept as low as possible. An increase in the polydispersity of the membrane can result from a micro phase separation of the DOPG and glycyrrhizin molecules within the membrane. Both molecules have significantly different dimensions along the membrane, which is why DOPG-rich membrane regions would have a larger membrane thickness than glycyrrhizin-rich regions. These locally different membrane thicknesses would finally lead to a higher membrane polydispersity.

## 4. Conclusions and Outlook

The influence of adding glycyrrhizin to long-time stable vesicles composed of the negatively charged phospholipid DOPG was investigated. Samples were prepared in a D_2_O-/H_2_O-based buffer solution with a pH value of 7.4, so that the lipid as well as the saponin should be deprotonated at their acidic functions. Stable SUVs were formed even at a glycyrrhizin content of up to 50 mol%. This was shown by cryo-TEM and small-angle scattering methods. The complementary usage of SANS and SAXS for samples prepared in D_2_O buffer showed the necessity of using both methods in the present case to derive profound structural parameters. Whereas from SANS data alone the influence of glycyrrhizin addition was only hardly resolvable on the membrane scale, SAXS data clearly indicated an incorporation of glycyrrhizin into the hydrophobic membrane part. In addition to a change in the membrane contrast, this incorporation causes a slight decrease in the membrane thickness accompanied by an increase in the membrane polydispersity. This increase in polydispersity might be a hint for a microphase separation of DOPG and glycyrrhizin within the membrane. Additional WAXS measurements indicated no change in the chain–chain correlation distance upon addition of glycyrrhizin. Comparably high amounts of glycyrrhizin are added and this observation might be explainable by similar molecular volumes of the entire glycyrrhizin molecule and the hydrocarbon region of DOPG. Even a microphase separation of lipid and saponin would not lead to an additional contribution in the WAXS signal and therefore this phase separation cannot be confirmed by the methods used in this study. The influence of the glycyrrhizin incorporation concerning a membrane pore formation or altering of the membrane elasticity should be further elucidated to gain a more precise picture of the possible mechanism of action. Possible methods for these investigations would be neutron spin echo experiments and/or a theoretical approach through molecular dynamics simulations. As already mentioned in the introduction, such mixed lipid glycyrrhizin vesicles might be useful in pharmaceutics.

## Figures and Tables

**Table 1:** Chain–chain correlation distances $d_{\mathrm{WAXS}}$ in vesicles composed of DOPG and glycyrrhizin with varying content obtained from the maximum of Lorentzian fits to WAXS data (see Figure 3) in D_2_O as well as H_2_O buffer. Within the experiment, no changes in $d_{\mathrm{WAXS}}$ were observed upon addition of glycyrrhizin.

$x_{\mathrm{glycyrrhizin}}$/mol%	$d_{(\mathrm{WAXS},D_{2}O)}$/Å	$d_{(\mathrm{WAXS},H_{2}O)}$/Å
0	4.50 ± 0.05	4.62 ± 0.04
1	4.51 ± 0.05	4.61 ± 0.03
5	4.52 ± 0.04	4.60 ± 0.03
10	4.50 ± 0.04	4.60 ± 0.03
20	4.47 ± 0.05	4.61 ± 0.04
30	4.50 ± 0.04	4.64 ± 0.03
40	4.52 ± 0.05	4.66 ± 0.03
50	4.53 ± 0.05	4.64 ± 0.03

**Table 2:** Core radii $R_{c}$ and corresponding polydispersities $\sigma_{R_{c}}$ of SUVs determined from CMS fits applied to SANS data of samples with different $x_{\mathrm{glycyrrhizin}}$ in D_2_O as solvent. Errors for $R_{c}$ result from the CMS fit with the program SASView [59] and seem to be underestimated in view of the high values of $\sigma_{R_{c}}$.

$x_{\mathrm{glycyrrhizin}}$/mol%	$R_{c}$/Å	$\sigma_{R_{c}}$/%
0	207 ± 2	37
1	208 ± 3	39
3	203 ± 3	42
10	202 ± 3	45
20	205 ± 3	48
30	176 ± 3	55
40	172 ± 3	61
50	156 ± 2	64

## Data Availability

The data will be available ond demand from the authors or after an embargo time of 3 years from the Institute Laue-Langevin (ILL) (doi:10.5291/ILL-DATA.9-11-1824).

## Supplementary Materials

The following are available online. Figure S1: Photographs of mixtures of DOPG and glycyrrhizin, Figure S2: Additional cryo-TEM images of DOPG and DOPG with 50 mol% glycyrrhizin, Figure S3: Comparison of X-ray scattering data for samples with different $x_{\mathrm{glycyrrhizin}}$ in D_2_O and H_2_O, Figure S4: Modified Kratky–Porod (MKP) plots for SAXS data in H_2_, Table S1: Total membrane thickness $d_{m,\mathrm{MKP}}$ derived from the MKP plot of SANS and SAXS, Table S2: Parameters to determine NSLDs and XSLDs of DOPG and glycyrrhizin, Table S3: Core radii $R_{c}$ obtained from CMS fits to SANS and SAXS data, Table S4: Scattering length densities for neutrons and X-rays for the hydrophobic membrane part, Table S5: Thickness of the hydrophobic membrane part $d_{\mathrm{tail}}$ obtained from CMS fits to SANS and SAXS data.

## Sample Availability

Samples of the compounds are not available from the authors.
