# Supplementary material for: Stable DOPG/Glycyrrhizin Vesicles with a Wide Range of Mixing Ratios: Structure and Stability as Seen by Scattering Experiments and Cryo-TEM
Source: Molecules. 2021 Aug 16;26(16):4959. doi: 10.3390/molecules26164959 (PMC8399256; doi:10.3390/molecules26164959)
Supplement: Supplementary file 1 [file molecules-26-04959-s001.zip › molecules-1301406-supplementary.pdf]

Electronic Supplementary Material for:

Stable DOPG/glycyrrhizin vesicles with a wide range of mixing ratios:

Structure and stability as seen by scattering experiments and cryo-TEM

Carina Dargel[1,♣], Friederike Gräbitz-Bräuer[1], Ramsia Geisler[1,‡], Pascal Fandrich[1], Yvonne Hannappel[1], Lionel Porcar[2] and Thomas Hellweg[1,*]

[1] Physical and Biophysical Chemistry, Bielefeld University, Universitätsstr. 25, 33615 Bielefeld, Germany
[*] E-mail: thomas.hellweg@uni-bielefeld.de, phone: +49 (0)521 1062055

[2] Institut Laue-Langevin - 71 avenue des Martyrs CS 20156, 38042 Grenoble Cedex 9 - France

♣ Current address: Institute of Physical Chemistry, University of Münster, Corrensstraße 28/30, 48149 Münster, Germany

‡ Current address: Medical Department 2, Hematology/Oncology and Infectious Diseases, University Hospital of Frankfurt, Frankfurt

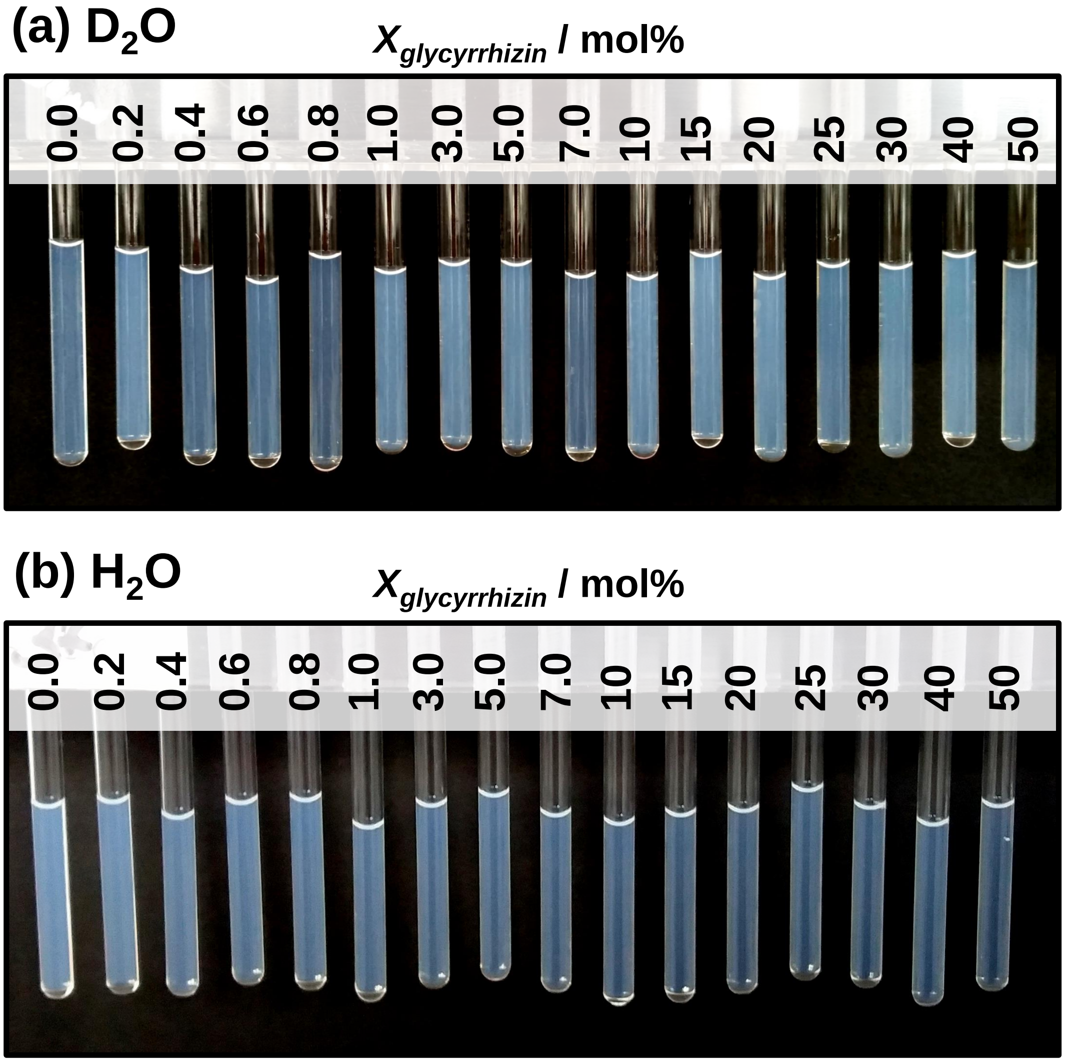

Figure S1. Photographs of mixtures of DOPG and glycyrrhizin in (a) D_2O and (b) H_2O at room temperature (≈25 °C). The samples were extruded (with increasing $x_{\text{glycyrrhizin}}$) through a membrane with a pore size of 500 Å. All samples exhibit a bluish color and are stable for at least six month. Bye bare eye no significant difference in appearance of the samples can be observed.

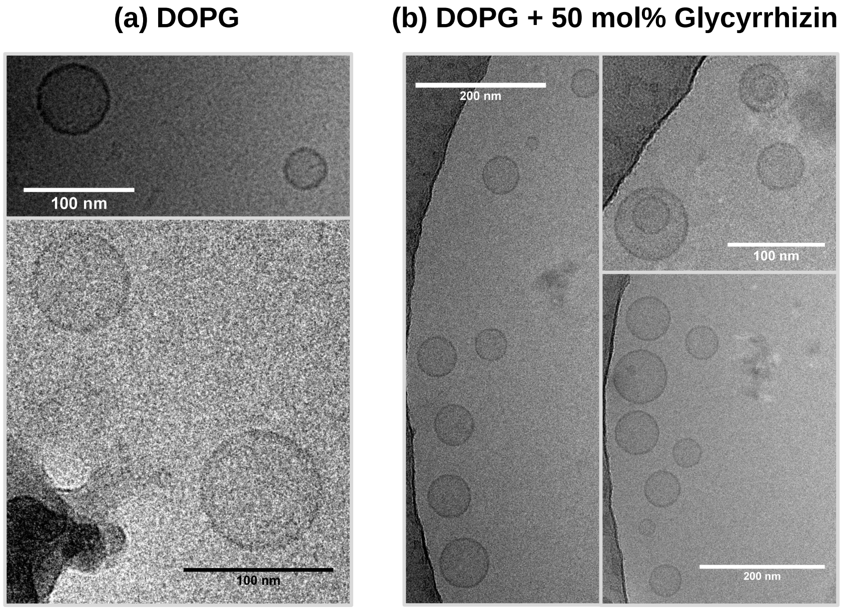

Figure S2. Cryo-TEM images of (a) DOPG and (b) DOPG with 50 mol% glycyrrhizin in H_2O buffer solution. The samples were diluted to a DOPG mass concentration of 0.5 g·L^{-1} and were kept at room temperature (≈25 °C) before freezing. In both cases, unilamellar vesicles are observed.

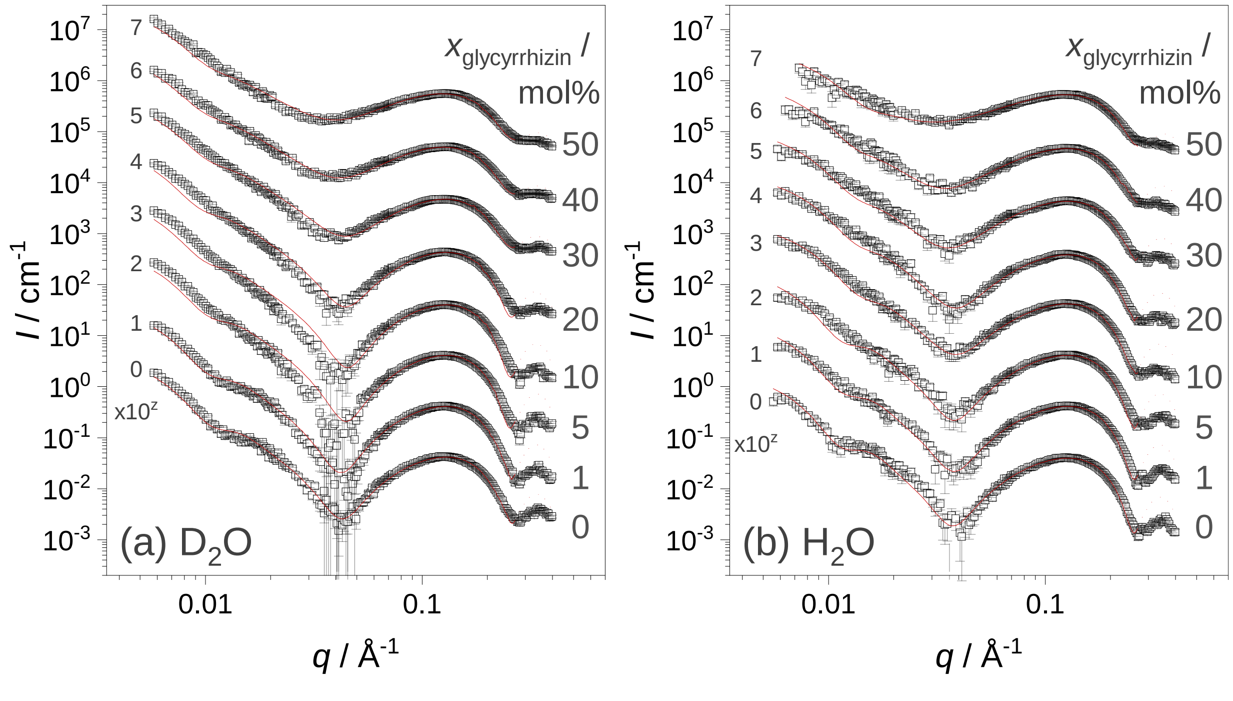

Figure S3. Comparison of X-ray scattering data for samples with different $x_{\mathrm{glycyrrhizin}}$ in (a) D_2O and (b) H_2O buffer as solvent. The glycyrrhizin content $x_{\mathrm{glycyrrhizin}}$ is denoted by the numbers on the right. For better readability, the data are scaled by different power of 10 indicated by the exponent z given as numbers on the left side of the graphs. Solid lines are CMS model fits from SASView [59].Dotted lines are extensions of the CMS fits beyond the fit range.

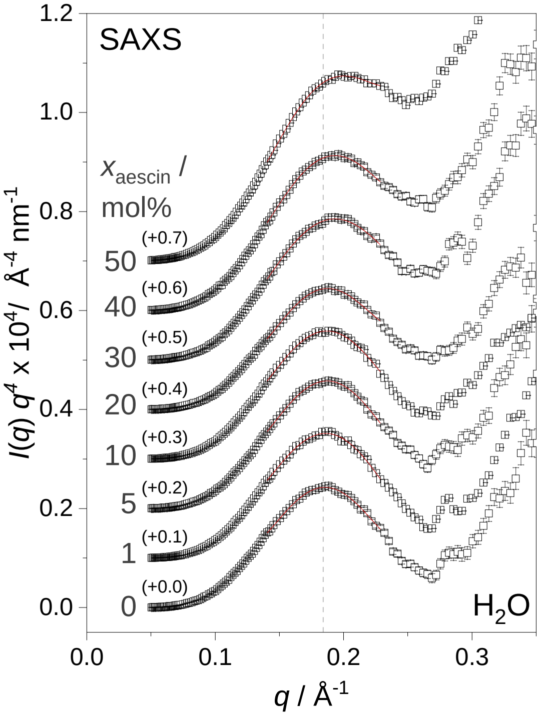

Figure S4. Modified K ratky-Porod (MKP) plots for SAXS data obtained for samples with different $x_{\text{glycyrrhizin}}$ in H_2O buffer as solvent[54]. Solid lines are 4th order polynomial approximations. The maximum of these approximations was used to determine the membrane thickness d_{MKP}. The glycyrrhizin content $x_{\text{glycyrrhizin}}$ is indicated by the numbers on the left. For better readability of the figure, t he d ata i s baseline-shifted by the numbers in brackets.

Table S1: Total membrane thickness d_{MKP} derived from the MKP plot of SANS and SAXS data of samples in D_2O and H_2O.

$x_{\text{glycyrrhizin}}$ mol%	$d_{\text{MKP,SANS,D}_2\text{O}}$ Å	$x_{\text{glycyrrhizin}}$ mol%	$d_{\text{MKP,SAXS,D}_2\text{O}}$ Å	$d_{\text{MKP,SAXS,H}_2\text{O}}$ Å
0	30.5 ± 0.3	0	33.3 ± 0.4	34.1 ± 0.4
1	30.9 ± 0.3	1	33.7 ± 0.4	34.0 ± 0.4
3	30.7 ± 0.3	5	33.7 ± 0.4	33.8 ± 0.4
10	30.4 ± 0.3	10	33.5 ± 0.4	33.8 ± 0.4
20	29.4 ± 0.3	20	33.0 ± 0.3	33.7 ± 0.4
30	29.6 ± 0.3	30	31.9 ± 0.3	32.7 ± 0.3
40	29.6 ± 0.3	40	31.7 ± 0.3	32.7 ± 0.3
50	28.0 ± 0.4	50	30.9 ± 0.3	31.6 ± 0.3

Table S2: Parameters to determine $NSLD$s and $XSLD$s of DOPG and glycyrrhizin at a temperature of 30 °C. M: molar mass, V_{mol}: molar volume, V: molecular volume, ρ: density.

	molecular formular	M g mol^{-1}	V_{mol} cm^3 mol^{-1}	V Å^3	ρ g cm^{-3}	$NSLD$ 10^{-6} A^{-2}	$XSLD$ 10^{-6} A^{-2}
DOPG	$C_{42}H_{78}O_{10}P$	773.53	761.78	1265.00 [53]	0.978	0.385	9.15
DOPG, head	$C_8H_{12}O_{10}P$	299.03	175.24	291.00 [53]	1.706	2.45	15.1
DOPG, tail	$C_{34}H_{66}$	474.50	586.54	974.00 [53]	0.809	-0.214	7.82
glycyrrhizin	$C_{42}H_{62}O_{16}$	822.93	572.6 [73]	950.85 [73]	1.44 [73]	1.48	13.2

Table S3: Core radii R_c obtained from CMS fits to SANS and SAXS data. In the case of samples in D_2O values were determined from SANS and in the case of H_2O values were derived from SAXS. In the latter case, the minimal q-value was not small enough for exact determination of R_c values and the uncertainty of these values might be larger than the depicted error. Values in brackets denote polydispersities of R_c (σ_{R_c}) in %.

$x_{\text{glycyrrhizin}}$ mol%	$R_{\text{c, SANS, D}_2\text{O}}$ Å	$x_{\text{glycyrrhizin}}$ mol%	$R_{\text{c, SAXS, H}_2\text{O}}$ Å
0	207 ± 2 (37)	0	204 ± 7 (32)
1	208 ± 3 (39)	1	191 ± 5 (36)
3	203 ± 3 (42)	5	172 ± 5 (38)
10	202 ± 3 (45)	10	142 ± 5 (46)
20	205 ± 3 (48)	20	138 ± 5 (50)
30	176 ± 3 (55)	30	131 ± 1 (56)
40	172 ± 3 (61)	40	124 ± 1 (59)
50	156 ± 2 (64)	50	108 ± 5 (60)

Table S4: Scattering length densities for neutrons ($NSLD_{\text{tail}}$) and X-rays ($XSLD_{\text{tail}}$) for the hydrophobic membrane part. Values were determined by fitting SAXS data of samples in D_2O and H_2O buffer by the CMS model. $NSLD$ values for samples in D_2O (indicated in italic letters) were calculated based on the corresponding $XSLD$ values. Additionally, calculated values for the head group region are listed for comparison and were taken from Table S2.

$x_{\text{glycyrrhizin}}$ mol%	$NSLD_{\text{tail, D}_2\text{O}}$ 10^{-6}Å^{-2}	$x_{\text{glycyrrhizin}}$ mol%	$XSLD_{\text{tail, D}_2\text{O}}$ 10^{-6}Å^{-2}	$XSLD_{\text{tail, H}_2\text{O}}$ 10^{-6}Å^{-2}
0	*-0.23 ± 0.01*	0	8.23 ± 0.01	8.18 ± 0.01
1	*-0.21 ± 0.01*	1	8.23 ± 0.01	8.19 ± 0.01
3	*-0.16 ± 0.01*	5	8.28 ± 0.01	8.18 ± 0.01
10	*-0.05 ± 0.01*	10	8.28 ± 0.01	8.18 ± 0.01
20	*0.11 ± 0.01*	20	8.24 ± 0.01	8.87 ± 0.01
30	*0.28 ± 0.02*	30	8.20 ± 0.01	8.11 ± 0.01
40	*0.37 ± 0.02*	40	8.09 ± 0.01	8.06 ± 0.01
50	*0.48 ± 0.02*	50	8.02 ± 0.01	7.89 ± 0.01
	$NSLD_{\text{head, D}_2\text{O}}$ 10^{-6}Å^{-2}		$XSLD_{\text{head, D}_2\text{O}}$ 10^{-6}Å^{-2}	$XSLD_{\text{head, H}_2\text{O}}$ 10^{-6}Å^{-2}
	2.45		15.1	15.1

Table S5: Thickness of the hydrophobic membrane part d_{tail} obtained from CMS fits to SANS and SAXS data of samples prepared in D_2O and H_2O buffer. Values in brackets denote polydispersities of d_{tail} ($\sigma_{d_{\text{tail}}}$) in %.

$x_{\text{glycyrrhizin}}$ mol%	$d_{\text{tail, SANS, D}_2\text{O}}$ Å	$x_{\text{glycyrrhizin}}$ mol%	$d_{\text{tail, SAXS, D}_2\text{O}}$ Å	$d_{\text{tail, SAXS, H}_2\text{O}}$ Å
0	26.3 ± 0.1 (-)	0	31.3 ± 0.1 (3)	31.0 ± 0.2 (0)
1	25.9 ± 0.1 (-)	1	31.4 ± 0.1 (1)	31.1 ± 0.2 (0)
3	25.9 ± 0.1 (-)	5	31.7 ± 0.1 (1)	31.0 ± 0.1 (0)
10	25.9 ± 0.1 (-)	10	31.5 ± 0.1 (4)	30.9 ± 0.2 (6)
20	25.9 ± 0.1 (-)	20	31.2 ± 0.2 (6)	30.9 ± 0.2 (4)
30	26.0 ± 0.1 (-)	30	30.8 ± 0.2 (10)	30.4 ± 0.2 (5)
40	26.0 ± 0.1 (-)	40	30.0 ± 0.1 (12)	30.1 ± 0.1 (7)
50	25.6 ± 0.1 (-)	50	29.7 ± 0.2 (13)	28.9 ± 0.2 (9)
